# Identif ication of duplicate accessions in the sweet maize collection by means of zein electrophoresis

**DOI:** 10.18699/VJ20.652

**Published:** 2020-10

**Authors:** V.V. Sidorova, Yu.A. Kerv, A.V. Konarev

**Affiliations:** Federal Research Centre the N.I. Vavilov All-Russian Institute of Plant Genetic Resources (VIR), St. Petersburg, Russia; Federal Research Centre the N.I. Vavilov All-Russian Institute of Plant Genetic Resources (VIR), St. Petersburg, Russia; Federal Research Centre the N.I. Vavilov All-Russian Institute of Plant Genetic Resources (VIR), St. Petersburg, Russia

**Keywords:** sweet maize, duplicate accessions, zein electrophoresis, protein markers, сахарная кукуруза, дублетные образцы, электрофорез зеина, белковые маркеры

## Abstract

Of all the subspecies of Zea mays L. cultivated in the world, sweet maize is the most important for the global economy. The leading seed-growing companies and research institutions around the world are engaged in breeding this crop. To meet the increasing demands of the industry to grain quality, it is important to select appropriate local varieties and lines for hybridization. Local (usually heterogeneous) varieties are a valuable source material for creating self-pollinated lines that contribute to a significant broadening of the genetic base of parental forms used in breeding. The advantages of sweet maize varieties and the interest of the food industry in them make it possible to consider accessions from the maize collection of the N.I. Vavilov Institute (VIR) as a potentially valuable source material for breeding. The present research concentrated on 19 local sweet maize varieties with different grain colors from the VIR collection, that is, 9 varieties with the blue color of ripe grain, 4 with white (colorless) grain, 3 with yellow, and 3 with red. The research included an analysis of zein electrophoretic patterns (protein markers); a study of their biotype composition and the nature of genetic polymorphism, as well as the creation of a protein pattern database for each accession. For a series of accessions with the same varietal name, but different catalog numbers, the degree of their identity was determined from their biotype composition in order to exclude duplication. Zein electrophoresis was carried out in vertical plates of 10 % polyacrylamide gel according to the standard ISTA technique developed with the participation of the Biochemistry and Molecular Biology Department of VIR. Zein patterns were used for the first time to electrophoretically study sweet maize varieties with different grain colors. Unique zein patterns were established for all the accessions studied, which makes possible their identification by specific marker components. The results of this work characterize zein electrophoresis as a useful tool for the identification and registration of duplicate accessions in the VIR collection of sweet maize varieties.

## Abstract (ru)

Из всех подвидов Zea mays L., возделываемых на земном шаре, самый значимый для мировой экономики – сахарная кукуруза. Ее селекцией занимаются ведущие семеноводческие фирмы и научные учреждения мира. Для удовлетворения возрастающих запросов производства к качеству зерна важное значение имеет правильный подбор местных сортов и линий для гибридизации. Местные (как правило, ге- терогенные) сорта – ценный исходный материал для создания самоопыленных линий, что способствует су- щественному расширению генетической базы используемых в селекции родительских форм. Достоинство сортов сахарной кукурузы и интерес к ним пищевой промышленности позволяют рассматривать генети- ческие ресурсы коллекции Всероссийского института генетических ресурсов растений им. Н.И. Вавилова (ВИР) в качестве потенциально ценного исходного материала для селекции. Целью нашей работы было выявление дублетных образцов в коллекции сахарной кукурузы ВИР. В задачи исследования входило: проведение скрининга 19 местных сортов подвида сахарной кукурузы из коллекции ВИР с разным цве- том зерна (9 стародавних местных с синей окраской зрелого зерна, 4 – с белой (бесцветной), 3 – с желтой и 3 – с красной окраской) по электрофоретическим спектрам зеина (белковым маркерам); изучение их биотипного состава и характера генетического полиморфизма, создание паспортной базы данных каждого изученного образца по белковым спектрам; для некоторых сортов, имеющих одинаковое сортовое название, но разные каталожные номера, установление степени идентичности их по биотипному составу с целью исключения дублетов. Электрофорез зеина проводили по стандартной методике ISTA, разрабо- танной с участием отдела биохимии и молекулярной биологии ВИР, в вертикальных пластинах 10 % по- лиакриламидного геля. Методом электрофореза по спектрам зеина впервые исследованы сорта подвида сахарной кукурузы с разной окраской зерна. Для всех изученных образцов установлена уникальность спектров зеина, что позволяет идентифицировать их по специфичным для них маркерным компонентам. Результаты настоящей работы свидетельствуют о перспективности использования электрофореза зеина для выявления, идентификации и регистрации дублетных образцов в коллекции сортов подвида сахарной кукурузы ВИР.

## Introduction

According to many researchers, Zea mays L. is the only species of the genus Zea L. unknown in the wild (Shmaraev, 1999; Matsuoka et al., 2002). The primary focus of primitive maize formation was the territory of Mexico (Piperno, Flannery, 2001; Wu, Messing, 2014), and the secondary one was the highlands of Peru (Zhukovsky, 1971). According to the taxonomy of the genus developed at VIR, sweet maize has been separated into the subspecies Zea mays L. subsp. saccharata (Sturt.) Zhuk. The su1 gene found in regular sweet maize ensures a high free sugar content at the expense of a reduced proportion of starch in the endosperm.

Supersweet maize has the sh2 gene in its genome, which is located on the third chromosome in the recessive state. Both su1 and sh2 genes affect the synthesis of carbohydrates in grain: su1 blocks the conversion of sugars into starch, and sh2 blocks the synthesis of starch during the conversion of sugars into dextrins. When su1 and sh2 are combined in the same genotype, the sugar content increases up to 21–35 %, while mature kernels look feeble and wrinkled (Suprunov et al., 2017). Currently, the world’s leading breeding and seed-producing companies are working on the creation of varieties of supersweet maize, which is not a genetically modified product; all its hybrids are produced by crossing plants and breeding for high sh2 values (Tracy, 1997).

In the Russian Federation, sweet maize breeding is carried out at the All-Russian Research Institute of Maize, the Kabardino-Balkarian Research Institute of Agriculture (Nalchik city), the Krasnodar Research Institute of Agriculture, the KOS MAIS Scientific and Production Association, and the Research Institute of Agriculture of the South-East. According to the State Commission of the Russian Federation for Testing and Protection of Breeding Achievements, 121 varieties of sweet maize have been registered and admitted for use in the Russian Federation (State Register for Selection Achievements, 2019).

At the stage of milky wax (technical) ripeness, sweet maize grain has a very tender pericarp, which is especially valuable for canning (Tanaboon, 1995). In terms of basic nutrients content, sweet maize keeps abreast of such nutritionally valuable vegetable legumes as green peas and green beans, and in terms of carbohydrate content it is significantly superior to them (Hooda, Kawatra, 2013). It is very important for the organization of a healthy diet that maize protein is much less allergenic than wheat protein (Holding, 2014). Besides, unlike other vegetable crops, sweet maize does not accumulate nitrates in kernels, and leaf wrapped around the cobs protect the grain from airborne pollution with various substances, including radionuclides. Sweet maize is also used for medicinal purposes. Extracts from maize flower parts (stigmas) are used in official and folk medicine for the treatment of inflammatory diseases of the liver and gall bladder (Kumar, Jhariya, 2013).

To meet the growing demands of the industry to grain quality, the proper selection of local varieties and lines for hybridization is important. Old (usually heterogeneous) varieties are a valuable source material for creating selfpollinated lines, which contributes to a significant broadening of the genetic base of parental forms used in breeding. The ripe kernels of various local varieties of sweet maize can have different colors, e. g., white (no color), yellow, brown, red, violet, blue, etc. Breeding for grain color is a result of the development of a new trend, that is, the aesthetic breeding (Novoselov, 2007).

The advantages of sweet maize varieties and the interest of the food industry in them make it possible to consider the germplasm available in the maize collection of the N.I. Vavilov All-Russian Institute of Plant Genetic Resources (VIR) as a potentially valuable source material for breeding. The collection acquires a higher significance with the increasing completeness of information about each accession conserved in it. In this regard, the identification of duplicate accessions gains importance as it helps to avoid expenses associated with studying the accessions identified as duplicates, as well as with their maintenance and storage.

At present, molecular (DNA and protein) markers are used along with morphological characters in order to control the genetic integrity (authenticity) of accessions, identify duplicates and reveal errors that can occur in the course of regeneration (Pyukkenen et al., 2005; Konarev, 2006; Potokina, 2009; Strelchenko, Kovaleva, 2009). Storage proteins should be recognized as more reliable for the purposes of seed control and solving a number of breeding problems. They are numerous, most polymorphic, and localized in morphogenetically homogeneous tissues, i. e., in the mature seed endosperm (Konarev, 1983). Protein markers make it possible to control the biotypic (genotypic) composition of a variety’s population – for example, to reveal a decrease in the population heterogeneity that leads to a deterioration of the adaptive properties of the variety (Konarev et al., 2000; Konarev, 2006). The analysis of grain storage protein polymorphism is the basis of the international and domestic standard methods for identification of lines and varieties (Cooke, 1978; Konarev et al., 1987). The present work employed zeins, the maize storage proteins, whose electrophoretic patterns are reliable markers for the varietal identification and maize genepool registration. Maize is a cross-pollinated plant, therefore zeins are characterized by a rather high polymorphism and are widely used in the study of maize genetic resources (Sidorova et al., 2012, 2015, 2018). When assessing the specificity of a variety from protein bands, the analysis of individual grains is required. The electrophoretic pattern of zein of a single grain marks the corresponding biotype (genotype).

The objective of the present study was to identify duplicate accessions in the sweet maize collection at VIR. The tasks set were as follows: to use electrophoretic patterns of zein to determine the biotype composition and character of polymorphism of differently colored sweet maize local varieties from the VIR collection; carry out their certification on the basis of protein bands; to use the biotype composition for establishing the degree of identity of some accessions with the same name but different catalog numbers in order to eliminate duplicates.

## Materials and methods

The work was carried out in the Department of Biochemistry and Molecular Biology of VIR. The material used for the study were the ripe kernels of 19 local sweet maize varieties with different grain colors (50 kernels per each accession), regenerated at the Volgograd Experiment Station of VIR (see Table).

**Table 1.**
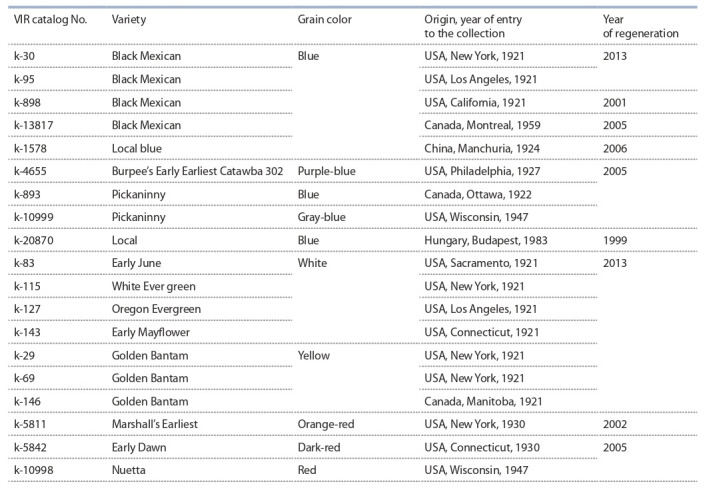
List of accessions used in the research

Zein electrophoresis was carried out in vertical PAAG plates without cooling for 4.5 h at a voltage of 500–580 V, according to the standard ISTA method developed with the participation of the Department of Biochemistry and Molecular Biology of VIR. The gel plates contained 10 % acrylamide and 8 M urea. Zein was isolated from single grains with a solution containing 6 M urea and 0.01 M dithiothreitol. The stained and dried gels with electrophoretic patterns were scanned. The registration of electrophoretic zein patterns was carried out using a standard, the selfpollinated F2 line from France. The numbering of protein components corresponds to the magnitude of their electrophoretic mobility (Kerv, Sidorova, 2018).

## Results and discussion

**Fig. 1.**
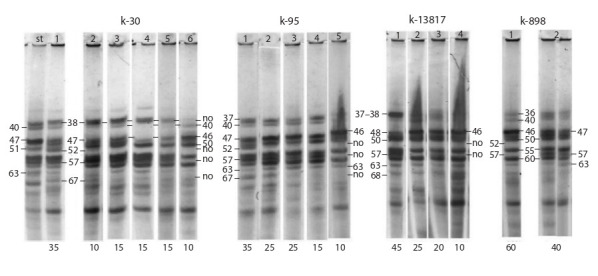
Zein electrophoregrams for the accessions of the sweet maize variety ‘Black Mexican’ with blue grain: k-30, k-95, k-13817 and k-898. Here and also in Fig. 2–6: the figures above the pattern indicate the biotype number, and those along the pattern indicate the numbers of polypeptides in the pattern. The figures under the patterns indicate the frequency of occurrence of each biotype.

Figure 1 shows zein electrophoretic patterns of four accessions of the sweet maize variety ‘Black Mexican’ with blue grain, registered in the collection in different years under different catalog numbers. The patterns of biotypes 3, 4 and 5 (with a frequency of occurrence of 15 %) lack the combination of components 52–67. Between themselves, they differ by the presence/absence of components 40, 47, and 63. Biotype 6 is rare (10 %); its patterns lack combinations of components 38–57 and 52–67.

Five biotypes were identified by zein patterns in the variety with the same name and the catalog number k-95. It does not have a basic pattern type. Biotype 1 and the frequency of occurrence (35 %) make accessions k-30 and k-95 identical. Biotype 2 of accession k-95 differs from biotype 2 of k-30 only by the absence of component 37 in its patterns, and by a higher frequency of occurrence (25 %). Biotypes 3 and 4 (k-95) have patterns that are different in composition and are not found in k-30. Biotype 5 is rare (10 %); it is identical in composition and frequency of occurrence to biotype 6 of k-30. On the basis of the foregoing, accessions k-30 and k-95 can be regarded as genetically close (due to the presence of the frequent biotype 1); however, they are not duplicates.

Unlike accessions k-30 and k-95, accessions k-13817 and k-898 have different compositions of zein patterns. They exhibit low intra-varietal polymorphism (four and two biotypes, respectively). Their patterns lack the combination of components 52–67, which obligatorily occurs in the patterns of the frequently encountered biotypes in accessions k-30 and k-95. Biotypes 2, 3, and 4 of k-13817 (with the total frequency of occurrence of 55 %) and biotype 1 in k-898 (60 %) do not have a combination of components 38–57 in the patterns. Such a biotype as the one in accessions k-30 and k-95 occurs rarely (10 %). The patterns of the frequently encountered biotype 1 in k-13817 (45 %) and biotype 2 in k-898 contain a combination of components 38–57 (40 %). However, these biotypes are not identical, since the intensity of the combination of components 38–57 is higher in k-13817 than in k-898. Also, the patterns of the accessions considered contain additional components. These types of patterns do not occur in k-30 and k-95.

All the accessions with the same varietal name have zein patterns that differ in component composition, which indicates that these accessions should be given different catalog numbers and stored separately.

**Fig. 2.**
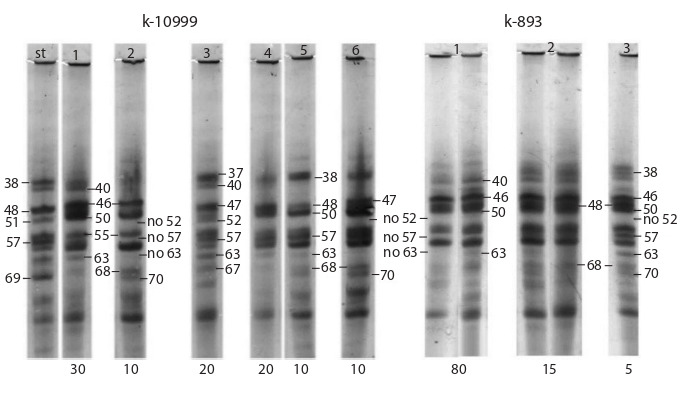
Zein electrophoregrams for the accessions of the sweet maize variety ‘Pickaninny’ with blue grain: k-10999 and k-893.

Figure 2 shows zein electrophoretic patterns of two accessions of the sweet maize variety ‘Pickaninny’ with blue grain, registered in the collection under different catalog numbers (k-10999 and k-893). Accession k-10999 is characterized by significant intra-varietal polymorphism. Six types of zein patterns with different frequencies of occurrence have been revealed. Biotype 1 occurs more often than the others (30 %). Its patterns have no combinations of components 38–57 and 52–67. Biotype 2 is rare (10 %), it is identical to biotype 1 in terms of the presence of intense components 46, 50, and 55 in the patterns, and differs from it by the absence of components 40 and 63, as well as by an additional component, 70. The patterns of the remaining biotypes (3–6) have a combination of components 38–57. Of these, only biotype 3 differs from the others by the presence of a combination of components 52–67 (20 %) in its patterns. The patterns of the remaining biotypes (4–6) are characterized by the presence/absence of a number of components with weak intensity. Accession k-893 of the old variety ‘Pickaninny’ differs from k-10999 by low intra-varietal polymorphism. Only three biotypes have been identified in it. The frequency of occurrence of the main biotype 1 is 80 %. The zein pattern makes it identical to biotype 1 of k-10999 (30 %). Its patterns lack a combination of components 38–57. Biotype 2 is less common (15 %); in terms of the presence of intense components in the patterns, it is identical to biotype 1, but differs from it by the presence of additional components with low intensity. The main biotypes 1 and 2 in k-893 are identical concerning the basic types of patterns 1 and 2 of k-10999. Biotype 3 is extremely rare (5 %). This type of pattern does not occur in k-10999.

**Fig. 3.**
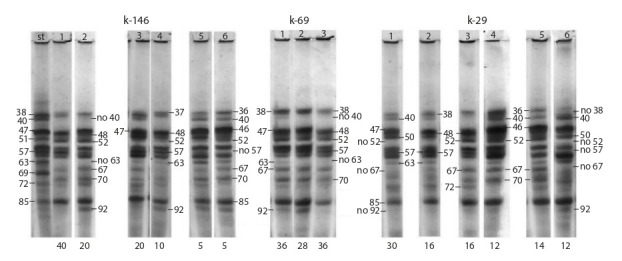
Zein electrophoregrams for the accessions of the sweet maize variety ‘Golden Bantam’ with yellow grain: k-146, k-69 and k-29.

The accessions with the same varietal name have zein patterns with different compositions and, therefore, they cannot be regarded as one and the same accession. Figure 3 shows zein patterns of three accessions of the sweet maize variety ‘Golden Bantam’ with yellow grain. The old accession k-146 showed significant intra-varietal polymorphism. Six pattern types have been identified in it. Patterns of all biotypes are characterized by the presence of a combination of components 52–67 with a varying degree of intensity. Four of them (1–4) also have a combination of components 38–57. Biotypes 5 and 6 are rare. Unlike the frequently encountered biotypes, they lack a combination of components 38–57 in their patterns. Biotypes 5 and 6 are rare. They differ from the frequently encountered biotypes by the absence of a combination of components 38–57 in their patterns. The frequently occurring biotypes 1 and 2 are distinguished by the patterns without components 40 and 63, which are quite intense in the patterns of rare biotypes 5 and 6. Biotypes 1–4 are characterized by different combinations of components 47, 48 and 50. Component 50 is absent in the patterns of biotypes 1 and 2.

Accession k-69 is characterized by low intra-varietal polymorphism. Three biotypes with the frequency of occurrence of 28–36 % have been identified in it. According to the pattern types, k-69 is close to biotypes 1, 2 and 3 of accession k-146. Biotypes 4, 5, and 6 in k-146 have compositionally different patterns, which are not found in k-69.

The third accession from the ‘Golden Bantam’ k-29 group is characterized by high intra-varietal polymorphism, and the number of the biotypes identified is six. The most common is biotype 1 (30 %); the frequency of occurrence of the remaining ones is approximately the same and equals 12–16 %. Only two biotypes (3 and 4) have patterns that are identical to those of biotypes 2 and 3 of accession k-146. Biotypes 1, 2, 5 and 6 from k-29 are absent in ‘Golden Bantam’ accessions k-146 and k-69, since their patterns do not have a combination of components 52–67, which is typical of all types of patterns of k-146 and k-69.

The old accessions with the same varietal name have zein patterns with different compositions, therefore, they are not duplicates.

**Fig. 4.**
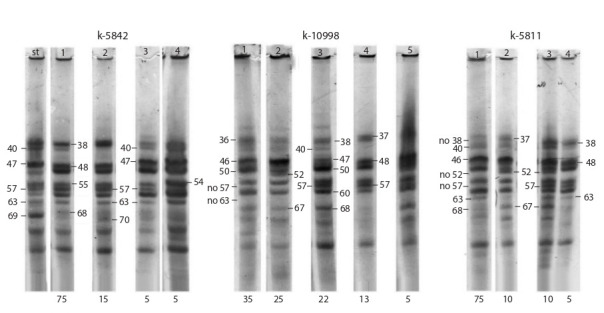
Zein electrophoregrams for the accessions of sweet maize varieties with red grain: k-5842, k-10998 and k-5811.

Figure 4 presents the electrophoretic zein patterns of three local sweet maize varieties with red grain. The accessions studied have individual zein patterns specific for each variety.

Accession k-5842 was found to have the main pattern type (biotype 1) with the frequency of occurrence of 75 %, and two rare ones (biotypes 3 and 4). Biotype 2 (15 %) differs from biotype 1 by the absence of component 40 in the patterns. Specific for this variety was the presence of the intense component 63 and a combination of components 38–57, which are present in the patterns of all biotypes. Also, the absence of a combination of components 52–67 in its patterns is specific to it.

Accession k-10998 is noted for high intra-varietal polymorphism. No main type of zein pattern was revealed for it. Biotype 1 with the 35 % frequency of occurrence, biotype 2 (25 %) and biotype 3 (22 %) are more common than the others. Specific to this variety is the absence of component 63 in the patterns of all biotypes. This distinguishes it from accessions k-5842 and k-5811 and increases the likelihood of obtaining a good hybrid combination with red grain.

Accession k-5811 is characterized by low intra-varietal polymorphism. The main pattern type (biotype 1) with the 75 % frequency of occurrence and three biotypes with a low frequency of occurrence (from 5 to 10 %) were revealed. The absence of the combinations of components 38–57 and 52–67 in the patterns of the main biotype turned out to be specific for it, as well as the presence of components 40 and 63 in the patterns of all biotypes.

It was found that all studied varieties with red grain color have specific components by which they can be identified, new hybrids can be created, and new lines can be selected on their basis.

**Fig. 5.**
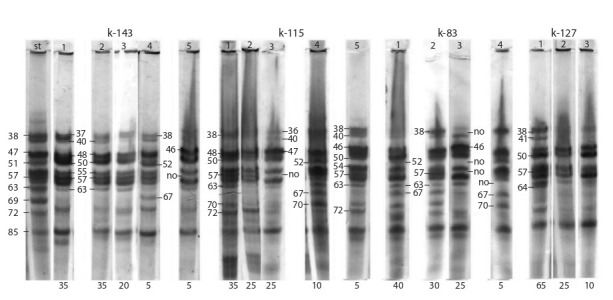
Zein electrophoregrams for the accessions of sweet maize varieties with white grain: k-143, k-115, k-83 and k-127.

Figure 5 demonstrates zein patterns of four sweet maize varieties with white grain. The accessions studied have individual specific patterns. Two accessions, k-143 and k-115, exhibit significant intra-varietal polymorphism. They have five types of patterns with different frequencies of occurrence. The broader the polymorphism of the varieties, the more difficult it is to identify their main pattern type. However, biotype 1 (35 %) and biotype 2 (35 %) are more common in k-143 than the others. They have identical pattern types and differ from each other in the intensity of manifestation of individual components. Biotypes 1 and 2 can be considered as the main patterns for k-143. Biotype 3 (20 %) differs from the first two types by the absence of component 40 in the patterns. Biotypes 4 and 5, which are rare, have compositionally different zein patterns. The combination of components 52–67 is intense in the biotype 4 patterns, while it is absent in patterns of the remaining biotypes. The biotype 5 patterns have no combination of components 38–57, which is specific for all biotypes of this variety.

Biotype 1 (35 %) occurs more frequently than the others in accession k-115. The type of this biotype pattern is unique for this accession, since it is not identical in component composition to other frequently occurring biotypes 2 and 3 (25 % each), moreover, to rare biotypes 4 and 5 (10 and 5 %, respectively). A low intensity of the zone of manifestation of components 36–40 is specific for this variety.

Biotype 1 (40 %) and biotype 2 (30 %) occur more frequently in accession k-83 than in the others. A combination of components 52–67 is manifested in the patterns of biotypes 1 and 2, though in biotype 2 it has low intensity. A combination of components 38–57 is intense in the biotype 2 patterns, whereas it is absent in the patterns of biotypes 1 and 3. Component 63, which is specific for the patterns of this variety, is absent in the patterns of rare biotype 4.

Accession k-127 is characterized by low polymorphism. The main biotype 1 with a 65 % frequency of occurrence, as well as biotypes 2 (25 %) and biotype 3 (10 %) were identified. This accession is unique among all the sweet maize varieties studied. The specific component 64 is present in its patterns. The absence of a combination of components 52–67 in the patterns of all biotypes was also specific to the variety. Therefore, there is a high degree of probability that this variety can be successfully used for creating new improved hybrids.

**Fig. 6.**
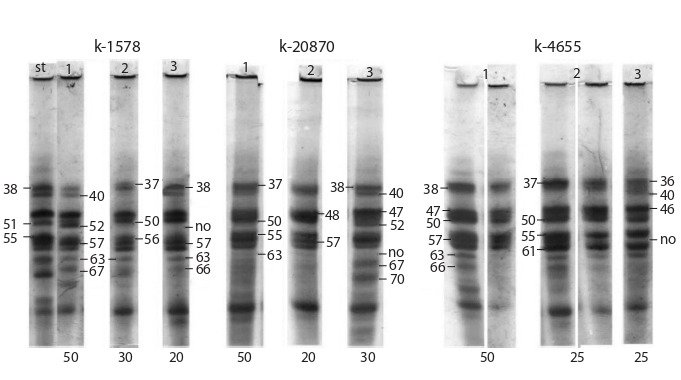
Zein electrophoregrams for the accessions of sweet maize varieties with blue grain: k-1578, k-20870 and k-4655.

Figure 6 presents the electrophoretic patterns of zein of three sweet maize varieties with blue grain. All accessions have different names and catalog numbers, and there are no low polymorphic varieties among them. No main type of pattern, the frequency of occurrence of which would be above 50 %, has been identified in them. However, the most common biotype amounts to 50 % in accessions k-20870, k-1578 and k-4655. The varieties studied have specific pattern types. Accession k-1578 is characterized by the presence of combinations of components 38–57 and 52–67 in the biotype 1 pattern, as well as of components 40 and 63. In contrast to biotype 1, the combination of components 52–67 and component 40 have a low intensity in biotype 2. A distinctive feature of biotype 2 is the presence of intense components 37 and 50 in the patterns. A combination of components 52–67, as well as components 37 and 47, are absent in the biotype 3 patterns. This biotype occurs less frequently than the others (20 %). The combination of components 38–57, as well as components 40 and 63, are specific to this variety.

Three biotypes have been revealed in accession k-20870. Biotype 1 (50 %) occurs more frequently than the others. The patterns of this biotype contain an intense combination of components 38–57, as well as intense components 37 and 47. Components 40 and 63 are characterized by low intensity. Biotype 2 with its 20 % frequency of occurrence differs from biotype 1 only by intense component 48 and a weak intensity of components 37, 40, and 63, as well as by the absence of component 47. In contrast to biotypes 1 and 2, the combination of components 52–67, as well as components 40 and 47, are intense in the patterns of biotype 3 with a 30 % frequency of occurrence. The presence of an intense combination of components 38–57 is specific to this variety, as well as the absence or a very weak intensity of component 63 in the patterns of all biotypes.

Three biotypes have been revealed in accession k-4655. Biotype 1 occurs most often and has a 50 % frequency of occurrence. A combination of components 38–57 is well manifested in zein patterns of biotype 1. In some patterns, this combination, as well as component 63, may have a low intensity. Biotype 2 differs from biotype 1 by a lower frequency of occurrence (25 %). The patterns of this biotype contain intense components 37 and 46, which are absent in the patterns of biotype 1. The combination of components 38–57 has a weak intensity in the patterns of biotype 2.

Unlike biotypes 1 and 2, biotype 3 (25 %) has different pattern compositions. A combination of components 38–57 is absent in the patterns of biotype 3. In contrast to biotypes 1 and 2, intense components 36 and 40 are present in the patterns. The absence of a combination of components 52–67 is specific to the variety, which is characteristic of other sweet maize varieties.

## Conclusion

Based on the above, it can be concluded that among the accessions with blue grain and the same varietal name of ‘Black Mexican’, two accessions, k-30 and k-95, can be regarded as genetically close varieties, though not as duplicates. Unlike k-30 and k-95, two other accessions, k-13817 and k-898, have low polymorphism and compositionally different pattern types. Two accessions with blue grain and the same varietal name of ‘Pickaninny’ have different VIR catalog numbers, k-10999 and k-893, and are not duplicates either. Accession k-10999 is characterized by significant polymorphism and has six biotypes. Accession k-893 has a low intra-varietal polymorphism (three biotypes) and demonstrates the absence of a significant number of biotypes that are characteristic of k-10999. The varieties with the same name of ‘Golden Bantam’ and yellow grain color (k-146, k-69 and k-29) were also found to contain no duplicates. Accessions k-146 and k-29 are characterized by high intra-varietal polymorphism and have different pattern types. Accession k-69 has low intra-varietal polymorphism. The three biotypes found in k-146 and k-29 are not present in k-69. The accessions with the same varietal name have zein patterns with different compositions, which is an evidence of a significant difference between them and the impossibility to merge them.

Three local varieties of sweet maize with red grain were studied and specific components determined for each variety. Two sweet maize accessions, k-143 and k-115, with white grain exhibit significant intra-varietal polymorphism. Specific components have been identified for them. Accession k-127 is characterized by low polymorphism. It is unique due to the presence of component 64 in its patterns. Three sweet maize varieties with blue grain, k-20870, k-1578 and k-4655, are highly polymorphic and have pattern types specific to each variety.

The results of the work performed show that it is quite promising to use zein electrophoresis for the identification, registration, and revealing of duplicate accessions in the collection of sweet maize varieties with different kernel colors.

## Conflict of interest

The authors declare no conflict of interest.
